# Polymorphisms of **β**1-Adrenoreceptor Gene and Cardiovascular Complications in Patients with Thyrotoxicosis

**DOI:** 10.1155/2014/402897

**Published:** 2014-05-25

**Authors:** A. Y. Babenko, E. N. Grineva, D. A. Savitskaja, E. N. Kravchuk, V. N. Solncev, A. A. Kostareva

**Affiliations:** ^1^Almazov Federal Medical Research Centre, Institute of Endocrinology, Saint Petersburg, Russia; ^2^Almazov Federal Medical Research Centre, Group of Medical Statistic, Saint Petersburg, Russia; ^3^Almazov Federal Medical Research Centre, Institute of Molecular Biology and Genetics, Saint Petersburg, Russia

## Abstract

Human cardiac **β**1-AR perform a crucial role in mediating the cardiostimulating effects of norepinephrine. Gly389Arg and Ser49Gly polymorphisms of **β**1-adrenoreceptors (**β**1-AR) can influence the cardiovascular prognosis. However, the possible effect of Gly389Arg and Ser49Gly polymorphisms on heart function in thyrotoxicosis has not been studied. We investigated the possible link between Gly389Arg and Ser49Gly polymorphisms and echocardiography parameters in 165 normotensive patients with a thyrotoxicosis without any cardiovascular disorders. Echo-CG was performed according to standard protocol before and during the thyreostatic treatment. Our data demonstrate that both Gly389Arg and Ser49Gly polymorphisms have very moderate influence on the risk of left ventricular hypertrophy and atrial fibrillation with no statistically significant effects on cardiac function and the development of cardiovascular complications.

## 1. Introduction

The search of genetic predictors that determine the course and prognosis of various disorders is one of the major tasks in the concept of translational medicine. In thyrotoxicosis (TT), identification of genetic predictors, defining remission, and the risk of cardiovascular complications are of a great importance. Candidates for genetic prediction are the genes regulated by triiodothyronine and playing a key role in changing of the myocardial contractility and arrhythmogenesis. Among them are type 2 deiodinase gene polymorphisms, affecting the severity of clinical manifestations and cardiac complications in thyrotoxicosis [1, 2]. Another candidate gene is *β*1-adrenergic receptor (*ADRB1*) mediating the sympathetic inotropic and chronotropic effects in the heart and arrhythmogenesis [3–5]. Since thyroid hormones (TH) are important elements of the regulation of *β*1-AR expression and the density of *β*1-AR in myocardium during TT is considerably increased, the polymorphism of* ADRB1* can have a significant impact on the development of cardiovascular complications [6, 7].

Arg389Gly genotype has 73% frequency in Caucasians and is associated with threefold increase in basal and stimulated adenylate cyclase activity and sensitivity to stimulation of *β*1-AR [8, 9]. Accordingly, genotype Gly389Gly carriers differ from subjects with genotype Arg389Gly in cardiovascular risk. However, the evaluation of* ADRB1* polymorphisms in cardiovascular diseases such as ischemic heart disease and arterial hypertension gave only modest results. In several studies, a link between the Arg389Gly polymorphism and resting heart rate (HR) and diastolic arterial pressure was reported, while in other studies only a slight correlation with the left ventricular hypertrophy was noted [10, 11]. Studies* in vitro *showed that Ser49Ser homozygous subjects have lower functional activity of adenylate cyclase compared to Gly49Gly homozygous subjects, but higher sensitivity to stimulation by isoproterenol [12]. In the other study, there was no difference in basal activity of adenylate cyclase, but higher sensitivity to stimulation by isoproterenol was observed [9]. Thus, Gly49Gly genotype was considered to have cardioprotective effect in this study. Additionally, Gly49Gly homozygotes were reported to have lower basal heart rate (HR) and arrhythmogenesis [13–17]. Several authors considered heterozygous genotype Ser49Gly to be one of the genetic predictors of initial or secondary atrial fibrillation (AF) [18, 19]. However, no relationship between Ser49Gly genotype and family history of AF was found [20].

The impact of these polymorphisms on HR, blood pressure, and echocardiographic (EchoCG) parameters was not studied in patients with Graves' disease and thyrotoxicosis (TT). The aim of our study was to investigate the influence of Arg389Gly and Ser49Gly polymorphisms of* ADRB1* on clinical and EchoCG parameters and arrhythmias in patients with Graves' disease (GD) and TT. The dynamics of these parameters after euthyroidism achievement was also evaluated depending on genotype.

## 2. Patients and Methods

The study was performed according to Helsinki declaration, the study protocol was approved by local Ethical Committee of Almazov Federal Medical Research Centre, and written informed consent was obtained from all subjects prior to investigation. We included 165 patients with GD according to the following criteria:

1. age 18–55,
2. thyrotoxicosis associated with Graves' disease at the primary examination.

Exclusion criteria were the following:

1. concomitant cardiovascular diseases that can cause persistent abnormal changes of Echo CG parameters (heart ischemic disease, hypertension, valve disease, nonthyrotoxic cardiomyopathy, heart failure, diabetes mellitus, obstructive lung disease, and nonthyrotoxic arrhythmias),
2. condition which has contraindications with long thyrostatic therapy (increase ALT or AST more 5-point normal range, hepatic or renal failure, and intolerance to thioamides),
3. chronic intoxication (alcohol, toxicomania),
4. pregnancy or plan of pregnancy,
5. amiodarone therapy.

Control group (CG) included 101 age- and gender-matched euthyroid blood donors (Table 1).

## 3. Study Design

The diagnosis of GD was confirmed by the presence of thyrotoxicosis, diffuse hyper functional goiter, presence of autoantibodies to thyroid stimulating hormone (TSH) receptor, and/or increased radioactive iodine uptake at the moment of examination or in anamnesis. Investigation included clinical examination (heart rate, blood pressure), level of TH and TSH, EchoCG, and ECG, genotyping. The thyrostatic therapy included thioamides (mercasolil) in dose 30 mg followed by dose decreased to 10 mg (supporting dose) after restoration of euthyroidism. Patients had initial metoprolol therapy within 3–5 weeks discontinued after normal heart rate and euthyroidism achievement. The patients were reexamined 1 year following the beginning of the therapy.

## 4. Methods

### 4.1. Hormonal Tests

Free thyroid hormones and antibody serum levels were measured by immune-enzyme assay using ACCESS 2 analyzer (Beckman Coulter, USA) and immunochemical test systems (UNICEL DXI 800 ACCESS, Beckman Coulter): free triiodothyronine (fT3, the normal range is 4.0–8.0 pmol/L), free thyroxin (fT4, the normal range is 10–25 pmol/L), and TSH (the normal range is 0.25–3.5 mU/L). The levels of common T3 (the normal range is 1.2–2.6 nmol/L) and common T4 (the normal range is 77–142 nmol/L) were detected by immunoenzyme assay (Beckman Coulter, USA).

### 4.2. Echocardiography

The echocardiography examination was performed on Sonoline GGOS (Siemens) and included the following parameters: left atrial diameter (LAD), left ventricular mass index (LVMI), interventricular septum (IVS), left ventricle posterior wall (PWT), left ventricular end-diastolic volume (LVEDV), relative wall thickness (RWT), and isovolumic relaxation time (IVRT).

Left ventricular mass was calculated according to the formula recommended by ASE-2005 [21]. For description of LV geometry, we used the classification of Ganau et al. [22]: normal geometry (NG); eccentric hypertrophy (EH); concentric remodeling (CR); concentric hypertrophy (CH).

### 4.3. DNA Isolation and Genotyping

DNA was isolated from 200 *μ*L volume of the peripheral blood by phenol-chloroform extraction. Genotyping was performed by real time polymerase chain reaction on Applied Biosystems 7500 Real Time PCR System using reaction premix purchased from “Applied Biosystems.”

### 4.4. Statistical Analysis

The results were expressed as frequencies, mean ±S.D., or median and percentiles 25–75 (P25–75). Allelic frequencies were determined by gene counting, and deviations from the Hardy-Weinberg equilibrium were verified using an *χ* ^2^ test. Clinical and laboratory data were addressed using *χ* ^2^ test, unpaired Student's *t*-test, Mann-Whitney *U*-test, ANOVA, Kruskal-Wallis* H*-test, and Fisher exact test. A two-tailed *P* < 0.05 was considered statistically significant, and all analyses were performed by STATISTICA 6.0 software package (StatSoft Inc., USA).

## 5. Results and Discussion 

### 5.1. Gly389Arg Polymorphism

Genotype distribution for Gly389Arg polymorphism is presented in Table 2. Hardy-Weinberg test revealed the shift of Hardy-Weinberg equilibrium in the group of patients with Graves' disease. Homozygote frequencies were below the equilibrium (for a genotype 1 equilibrium level—12%, and for a genotype 3—44%) and heterozygote frequency, accordingly, was above equilibrium (equilibrium level—45%). The prevalence of different genotypes in Graves' disease slightly differed in our groups compared to European population but was similar in control group: CC—55%, CG—37%, and GG—8%, and the alleles frequencies were 0.26 for G allele and 0.74 for C allele. Allele G prevalence in patients with thyrotoxicosis was significantly higher compared to control group (*P* = 0.01) (Table 2).

We performed comparative analysis of clinical and laboratory parameters in patients with thyrotoxicosis depending on genotype (Table 3(a)). During the first examination, there was no significant difference in blood pressure (BP) and heart rate (HR); however, there was a tendency to lower diastolic BP in patients with GG genotype. There was no difference in other parameters that may affect the development of cardiovascular complications (age, duration of thyrotoxicosis, and level of thyroid hormones) between the groups (Table 3(a)). However, the multivariate analysis including these parameters as covariants was performed and did not show the significant difference.

The EchoCG parameters did not differ significantly in groups with different genotype. Left ventricular hypertrophy (LVH) was detected in 11% of patients in group 1 (GG), 26.5% of patients in group 2 (CG), and 22.6% of patients in group 3 (CC). Interestingly, concentric LVH was not observed in patients with GG genotype. The prevalence of pulmonary hypertension was measured in 107 patients and was confirmed in 60 (56%). The mean values of pulmonary hypertension did not differ between the genotype groups. Clinical investigation was repeated in all patients after a year of thyrostatic therapy and euthyroidism. No significant difference in blood pressure, heart rate, and TH was revealed (see Table 3(b)).

In EchoCG parameters, there was multidirectional dynamics of LVMI in allele GG carriers compared with allele CC carriers (Table 4).

Follow-up examination after one year showed the change in patients groups with different type of LV geometry: in GG group LVM was normalized, in CC group LV hypertrophy reduced to 21%, and in heterozygous group the number of patients with LV hypertrophy increased to 27.5%. Multidirectional dynamics for these groups was presented in Table 4.

The evaluation of rhythm disturbances associated with thyrotoxicosis showed that AF was not present in GG genotype carriers; heterozygous patients had AF in 19% (18/94) and homozygous CC carriers in 9.7% (6/62). Supraventricular extrasystoles were also less frequent among GG genotype patients (GG genotype—30%, GC genotype—57%, and CC genotype—42%).

In summary, analysis performed for Gly389Arg polymorphism did not reveal any significant difference in the parameters studied but showed a tendency for lower frequency of LV hypertrophy and absence of concentric hypertrophy in GG genotype carriers. The tendency for higher frequency of AF was observed in heterozygous genotype carriers.

### 5.2. Ser49Gly Polymorphism

Genotype distribution for Ser49Gly polymorphism is presented in Table 5. The genotype prevalence corresponded with Hardy-Weinberg equilibrium. There was no significant difference in the genotype prevalence compared to age- and gender-matched euthyroid patients (control group) (Table 5).

The clinical characteristic of patients with Graves' disease and thyrotoxicosis according to genotype is presented in Table 6. There was no difference in parameters that may affect the development of cardiovascular complications (age, level of free thyroid hormones, and duration of thyrotoxicosis) between the groups.

The EchoCG parameters were compared in different genotypes groups. In patients with GG genotype, a tendency to more prominent LV hypertrophy (*P* = 0.06) was noted when we compare all three genotypes and when we compare A allele with GG genotype (*P* = 0.02) (Table 7).

Due to the correlation of GG genotype of* ADRB1* Ser49Gly variant with LVM, we performed more detailed analysis of LV geometry in this group. In patients with GG genotype, LV hypertrophy was observed in 55.6% (33.4% - EH, 22.2% - CH), AA genotype carriers had LV hypertrophy only in 15.5% (EH—10.5%, CH—5%), and heterozygous patients had LV hypertrophy in 26% (EH—20%, CH—6%). There was no significant difference in CH and EH, but tendency for less frequency of LVH was in AA genotype carriers (*P* = 0.09).

Pulmonary hypertension was detected in 75% of GG carriers, 55.9% of AG carriers, and 56.7% of AA carriers. There was no difference in the presence and degree of pulmonary hypertension between various genotypes.

Follow-up examination after one year showed no significant difference in blood pressure, heart rate, and TH dynamics between genotype groups (Table 6(b)). At follow-up, the initially described difference in LVMI between carriers of different genotypes did not remain. The difference in T4 level could not influence the above parameters since all genotype carriers had normal T4 level. There was no difference in atrial arrhythmias between patients with various Ser49Gly genotypes.

There was no difference in atrial arrhythmias between patients with various Ser49Gly genotypes. In summary, the carriers of AA genotype of polymorphism of Ser49Gly had lower frequency of LV hypertrophy.

We also analyzed the possible effect of haplotype combination in Ser49Gly and Arg389Gly polymorphisms on clinical parameters and frequency of LVH and AF in patients with TT. There was no statistically significant effect of haplotypes on any of the clinical parameters analyzed, in spite of several tendencies which did not reach the significance level (Table 8).

Our study revealed no significant difference in heart rate and blood pressure level in patients with thyrotoxicosis carrying different genotypes in polymorphisms Gly389Arg and Ser49Gly of* ADRB1*. These results concur with the majority of studies that investigated these parameters in other cardiovascular disorders. Earlier, analysis of heart rate during stress echocardiography and heart rate in patients with hypertension gave no evidence linking heart rate with different* ADRB1* genotypes [23–25]. In contrast, the studies of larger number of patients gave the opposite results. Thus, Ser49Gly polymorphism had a significant effect on resting heart rate and patients with Ser49Ser genotype had higher average heart rate [26]. The same was noted also in Finnish study, including more than 800 patients [27]. Thus, we cannot exclude the fact that the lack of such correlation in our study is related to the small group size.

Our data demonstrate that both polymorphism Gly389Arg and polymorphism Ser49Gly have a very moderate tendency to influence the LV hypertrophy risk. While in several studies the association of LV hypertrophy with CC genotype in polymorphism Gly389Arg was confirmed, there are no data on Ser49Gly and the risk of LVH. According to well-known influence of heart rate on LV hypertrophy, such correlation would be logical.

The separate analysis of polymorphisms Gly389Arg and Ser49Gly did not show a significant difference in blood pressure level and the risk of AF between various genotype carriers. Some studies reported that AA genotype (Ser49) carriers of polymorphism Ser49Gly have a lower heart rate and lower frequency of rhythm disturbances and therefore lower mortality [19]. However, in Russian study, in which the prevalence of primary and secondary AF in carriers of different Ser49Gly genotypes was examined, the opposite data were obtained. Patients with primary AF and their relatives had more frequent heterozygous genotype Ser49Gly [18]. In case of secondary AF (ischemic heart disease and arterial hypertension), there was also a significant prevalence of heterozygous genotype carriers (Ser49Gly). The authors conclude that heterozygous genotype Ser49Gly may be one of genetic predictors for development of both primary and secondary AF. These differences can be explained by two factors: firstly, a significant difference in the frequency of polymorphic genotypes in different populations and, secondly, difference in AF etiology. There is a high probability that in patients with secondary AF the contribution of other factors significantly dominates genetic influences.

A significant limitation of this study is the small size of the groups. There is a possibility that the accumulation of additional data will reveal more significant differences. Further analysis on larger population of patients is needed.

## Figures and Tables

**Table 1:** Characteristics of the study groups of patients with Graves' disease and thyrotoxicosis and in control group.

Characteristics	Graves' disease, *n* = 165	Control group, *n* = 101
Age, years	42.5 ± 9.2	43.4 ± 14.3
Sex, male/female	23/142	20/81
Heart rate, bpt/min	95.9 ± 19.1*	72.6 ± 8.7
BP, mmHg	129.4 ± 15.9/75.8 ± 9.4	127.3 ± 9.7/72.4 ± 7.1
FT3, pmol/L	15.0 ± 9.1*	1.8 ± 1.2
FT4, pmol/L	45.4 ± 23.0*	14.2 ± 4.6
T3, total, nmol/L	5.6 ± 2.66	1.6 ± 0.81
T4, total, nmol/L	218.2 ± 69.44	127.8 ± 37.34
TSH, mMЕ/L	0.014; (0.010; 0.058)*	1.2; (0.6; 3.1)

GD: Graves' disease group, CG: control group, BP: blood pressure, FT3: free triiodothyronine, FT4: free thyroxin, and TSH: thyroid stimulating hormone.

*P < 0.0001 compared with control group.

**Table 2:** Genotype and allele distribution for Gly389Arg polymorphism in patients with Graves' disease and control group.

Genotype		Number of subjects, *n*	%	Allele frequency
Graves' disease group 165				
Group 1	GG (Glu389Glu)	9	5.4	A—0.66 G—0.34*
Group 2	CG (Glu389Arg)	94	57	
Group 3	CC (Arg389Arg)	62	37.6	

Control group 101	GG (Glu389Glu)	8	8	A—0.74 G—0.26
	CG (Glu389Arg)	37	37	
	CC (Arg389Arg)	56	55	

*P = 0.01 Graves' disease group versus control group.

**Table:** (a)

Variables	CC, *n* = 62	CG, *n* = 94	GG, *n* = 9
Sex, male/female	F: 56 (39.4%) M: 6 (26%)	F: 78 (55%) M: 16 (69.6%)	F: 8 (5.6%) M: 1 (4.4%)
Age, years	43.3 ± 9.5	42.1 ± 8.9	40.5 ± 9.8
Duration of thyrotoxicosis, month	58; 10; (6; 24)	90; 10; (7; 20)	8; 7; (5.5; 10)
HR, beats/min	92 ± 12	99 ± 23	90 ± 6
BP, systolic, mmHg	129 ± 16	129 ± 15	134 ± 20
BP, diastolic, mmHg	76 ± 10	76 ± 9	77 ± 12
FT3, pmol/L	20; 12.7; (7.2; 22.3)	37; 11.5; (9.5; 20.7)	5; 10.4; (9.7; 22.1)
FT4, pmol/L	27; 37.2; (30.4; 54.5)	45; 46.0; (28.0; 57.8)	7; 31.0; (29.4; 92.4)
T3, total, nmol/L	34; 4.8; (3.9; 6.8)	51; 5.2; (3.7; 7.8)	5; 5.8; (3.0; 5.9)
T4, total, nmol/L	36; 198; (165; 239)	49; 222; (187; 288)	3; 174; (133; 214)

**Table:** (b)

Variables	CC, *n* = 62	CG, *n* = 94	GG, *n* = 9
HR, beats/min	75 ± 13	79 ± 12	75 ± 4
BP, systolic, mmHg	121 ± 14	118 ± 13	122 ± 14
BP, diastolic, mmHg	73 ± 7	70 ± 9	69 ± 10
FT3, pmol/L	10; 4.1; (2.3; 7.5)	13; 6.2; (5.7; 7.9)	3; 3.7; (2.0; 5.4)
FT4, pmol/L	24; 14.0; (9.3; 20.1)	45; 14.4; (10.1; 19.6)	3; 19.4; (16.7; 27.1)
T3, total, nmol/L	9; 2.5; (1.8; 3.9)	25; 2.0; (1.6; 2.7)	2; 2.0; (1.5; 2.4)
T4, total, nmol/L	43; 116; (110; 136)	49; 99; (79; 127)	6; 81; (28; 135)

**Table 4:** Echocardiographic parameters in patients with thyrotoxicosis depending on genotype (Gly389Arg) after treatment.

Group	LVMI, g/m²	LVMI 2, g/m²	The difference between two assessments
1 gr. (GG), *n* = 9	96.6 ± 8.06	88 ± 6	−35.5; (−60; −11)
2 gr. (CG), *n* = 94	99.9 ± 2.69	102.6 ± 2.49	81; −1; (−11; 10.6)
3 gr. (CC), *n* = 62	99.4 ± 2.39	102 ± 4.32	35; 0; (−16; 4)
*P* value versus GG genotype	0.79	0.76	0.03

**Table 5:** Genotype and allele distribution for Ser49Gly polymorphism in patients with Graves' disease and control group.

Group		*n*	%	Allele frequency
Patients with thyrotoxicosis, *n* = 165	GG (Gly49Gly)	**9**	5.5	G—0.22 A—0.78
	AG (Ser49Gly)	**56**	33.9	
	AA (Ser49Ser)	**100**	60.6	

Control group, *n* = 101	GG (Gly49Gly)	**2**	2	G—0.17 A—0.83
	AG (Ser49Gly)	**31**	30.7	
	AA (Ser49Ser)	**68**	67.3	

**Table:** (a)

	AA, *n* = 100	AG, *n* = 56	GG, *n* = 9
Sex, male/female	F: 88 (62%) M: 12 (52.2%)	F: 46 (32.4%) M: 10 (43.5%)	F: 8 (5.6%) M: 1 (4.3%)
Age, years	42.1 ± 8.7	42.6 ± 10.3	45.7 ± 5.0
Thyrotoxicosis duration, month	93; 10; (6; 20)	54; 12; (6; 20)	9; 10; (8; 24)
HR, beats/min	95 ± 20	97 ± 17	92 ± 12
BP, systolic/diastolic, mmHg	129 ± 16/75 ± 9	130 ± 15/77 ± 10	130 ± 20/75 ± 9
FT3, pmol/L	39; 11.1; (9.0; 22.1)	20; 12.7; (9.7; 21.6)	3; 10.0; (9.0; 29.5)
FT4, pmol/L	47; 46.0; (30.5; 61.0)	28; 36.6; (26.0; 54.4)	4; 42.0; (23.8; 50.3)
T3, total, nmol/L	52; 5.2; (4.0; 7.9)	31; 4.1; (3.3; 6.8)	7; 5.2; (4.3; 9.0)
T4, total, nmol/L	54; 215; (185; 284)	29; 202; (164; 249)	5; 134; (116; 149)*

*P = 0.04 for GG versus allele A.

**Table:** (b)

	AA, *n* = 100	AG, *n* = 56	GG, *n* = 9
HR, beats/min	78 ± 14	77 ± 8	72 ± 12
BP, systolic/diastolic, mmHg	119 ± 14/70 ± 8	120 ± 13/71 ± 8	117 ± 16/73 ± 10
FT3, pmol/L	13; 6.0; (3.4; 7.9)	11; 5.4; (3.7; 7.7)	2; 2.3; (2.0; 2.6)
FT4, pmol/L	56; 15.0; (9.7; 25.0)	31; 11.4; (9.2; 17.0)	5; 19.4; (19.0; 19.4)
T3, total, pmol/L	24; 2.0; (1.6; 2.7)	11; 2.2; (1.4; 3.8)	1; 6.2;
T4, total, pmol/L	44; 107; (83; 137)	30; 104; (74; 116)	4; 119; (110; 128)*

*P = 0.01 for GG versus allele A.

**Table 7:** Echocardiographic parameters in patients with Graves' disease and thyrotoxicosis with different Ser49Gly genotypes.

	AA, *n* = 100	AG, *n* = 56	GG, *n* = 9	AA + AG
LVMI, g/m²	100.2 ± 22.1	97.0 ± 26.8	118.9 ± 44.9*	99.1 ± 23.8**

*P = 0.06 for AA and AG versus GG genotype.

**P = 0.02 for A allele versus GG genotype.

**Table 8:** The frequency of atrial fibrillation in different combination of genotypes in observed polymorphisms of the gene encoding *ADRB1. *

Genotype combination Ser49Gly/Glu389Arg	AF absence	AF presence	Overall
AG/GG	18; 78.3%	5; 21.7%	23
GG/CC	34; 97.1%	1; 2.9%	35
AG/CG	25; 89.3%	3; 10.7%	28
AA/CG	50; 79.4%	13; 20.6%	63

Overall	127; 85.2%	22; 14.8%	149
